# Supplementary material for: The Role of Gorse (Ulex parviflorus Pourr. Scrubs) in a Mediterranean Shrubland Undergoing Climate Change: Approach by Hyperspectral Measurements
Source: Plants (Basel). 2023 Feb 15;12(4):879. doi: 10.3390/plants12040879 (PMC9967484; doi:10.3390/plants12040879)
Supplement: Supplementary file 1 [file plants-12-00879-s001.zip › plants-2189705-supplementary.pdf]

plants

MDPI

Supplementary Information

The Role of Gorse (*Ulex parviflorus* Pourr. Scrubs) in a Mediterranean Shrubland Undergoing Climate Change: Approach by Hyperspectral Measurements

Audrey Marteau †, Martin Fourmaux † and Jean-Philippe Mevy *

IMBE-UMR CNRS 7263/IRD 237, Aix Marseille University, Avignon University, 13331 Marseille, France
* Correspondence: jean-philippe.mevy@univ-amu.fr; Tel.: +33-0413550766
† These authors contributed equally to this work.

Supplementary Table S1. Wavelengths (nm) that discriminate *Ulex parviflorus* from *Quercus coccifera, Cistus albidus* and *Rosmarinus officinalis.* ANOVA with *p-value <0.001* and post hoc analysis (Fischer's LSD test) from pairwise comparison in monospecific stands.

Near infrared (nm)	Shortwave of near infrared (nm)
755	1016
793	1044
800	1063
803	1107
829	1166
836	1186
840	1192
840	1192
873	1198
875	1207
882	1210
896	1218
922-923	1296
945	1304
950	1309
963	
989	

Supplementary Table S2. The most significant wavelengths between control (c) and rain exclusion treatment (e) for the different assemblages of *Cistus albidus* (Ca), *Rosmarinus officinalis* (Ro) and *Quercus coccifera* (Qc) in the presence of *Ulex parviflorus* (Up). ANOVA, $p < 0.05$. NS: non-significant.

	Visible (350-700 nm)	NIR (701-1350 nm)	SWIR1 (1351-1800 nm)	SWIR2 (1801-2350 nm)
cCa vs. eCa	NS	NS	NS	NS
cCaUp vs. eCaUp	350-351, 357, 361-363, 368, 371, 374, 376, 379, 380-382, 384, 391, 394, 398, 408, 411, 418-419, 430, 443, 457, 468, 492, 495, 508, 518, 521, 527, 529, 533-535, 546, 549, 557563, 568, 572, 574-576, 578-579, 590, 596, 600, 606, 608-610, 618-619, 633, 636, 639, 642, 653, 679, 690, 693, 699	705-706, 711, 719, 755, 783, 796, 798, 810, 840 849, 857, 863, 876, 891, 894, 898, 922, 941, 943-944, 951, 953, 960, 984, 1022, 1030, 1050, 1082, 1085, 1099, 1118-1119, 1127, 1129, 1140 1144-1145, 1149, 1153-1154, 1162, 1164, 1188, 1201, 1206, 1210, 1212, 1220, 1229, 1231-1232, 1235-1236, 1239, 1264, 1275-1276, 1281, 1289, 1301, 1308, 1314, 1316, 1320, 1328, 1337	1523, 1546, 1561, 1574, 1580, 1582, 1591, 1606-1607, 1645, 1659, 1666, 1668, 1670, 1683-1685, 1692, 1725-1726, 1729, 1743, 1749, 1754, 1761, 1763, 1780, 785, 1792-1793, 1795	2066, 2091, 2122, 2153, 2144, 2183, 2192, 2217, 2227, 2229, 2247, 2251, 2256, 2259, 2264, 2301, 2321, 2351, 2383,

cCaQcUp vs. eCaQcUp	423, 691	NS	1500	1993
cCaRoUp vs. eCaRoUp	NS	1254	NS	NS
cCaQcRoUp vs. eCaQcRoUp	487, 516, 590, 610 613, 636,	711, 715, 725, 752, 756, 791, 873, 1048, 1075, 1084, 1093, 1184	1573, 1601	2148
cQc vs. eQc	NS	NS	NS	NS
cQcUp vs. eQcUp	NS	NS	NS	NS
cQcCaUp vs eQcCaUp	350-700	701-1339	1431-1800	1801-1809, 1981-2399
cQcRoUp vs eQcRoUp	350-431, 436-437, 442-654, 676, 695	875, 890-1336	1490, 1506, 1511, 1527, 1537, 1541, 1547-1550, 1755, 1569-1570, 1583-1599-1709, 1721-1800	1805-1807, 2039, 2092, 2162, 2164, 2170, 2181, 2185-2186, 2202, 2213, 2219-2220, 2231, 2250, 2257, 2285, 2327
cQcCaRoUp vs eQcCaRoUp	351-352, 354, 357, 359-402, 408, 420, 433, 446, 452, 456-457, 463, 495, 505, 536, 544, 555, 557, 587, 590, 604, 612, 615, 625, 628-633, 661, 689, 697.	NS	NS	NS
cRo vs eRo	NS	NS	NS	NS
cRo-Up vs eRo-Up	533, 566	756, 780, 789, 817 850, 985, 1225, 1228 1248, 1279, 1297,	1714, 1779	NS
cRo-Ca-Up vs eRo-Ca-Up	NS	864, 885, 902, 909, 912, 922, 946, 960, 996, 1006, 1046, 1078, 1311	NS	NS
cRo-Qc-Up vs eRo-Qc-Up	350-404, 407-517, 522-534, 552, 557, 595, 599, 610, 626, 634-639, 645, 649, 651, 655-656, 659, 663-664, 667, 671, 673, 677-678, 681, 684-685, 689, 692, 695-696	701-702, 704, 713, 719, 726, 728, 732, 736, 738, 743, 749, 750-751, 755-756, 758-1000, 1001-1350	1351-1800	1801-1808, 1810-2399
cRo-Qc-Ca-Up vs eRo-Qc-Ca-Up	351-700	701-750, 756, 766, 769, 772-1339	1431-1800	1803-1809, 1981-2399

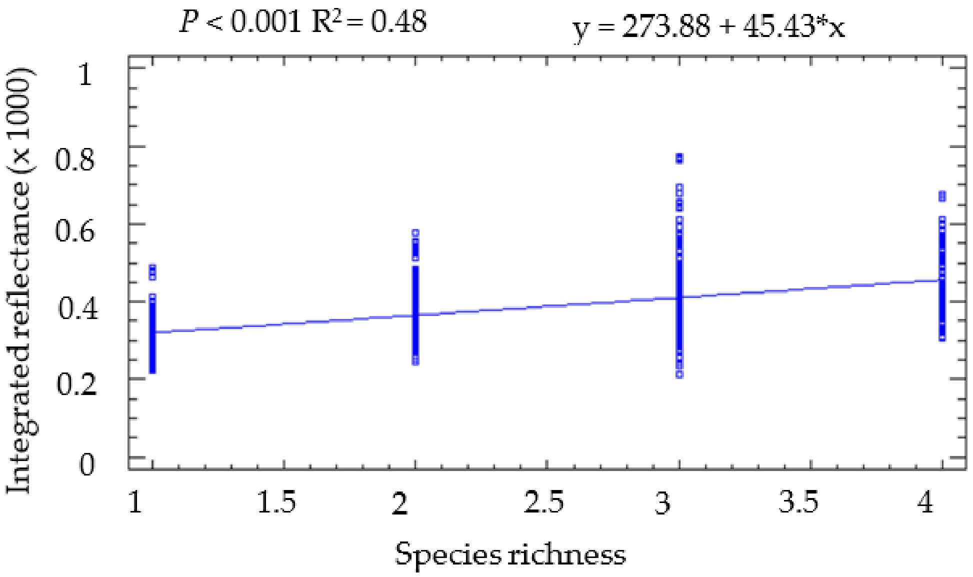

Supplementary Figure S1. Linear model of the integrated reflectance versus species richness in control condition.

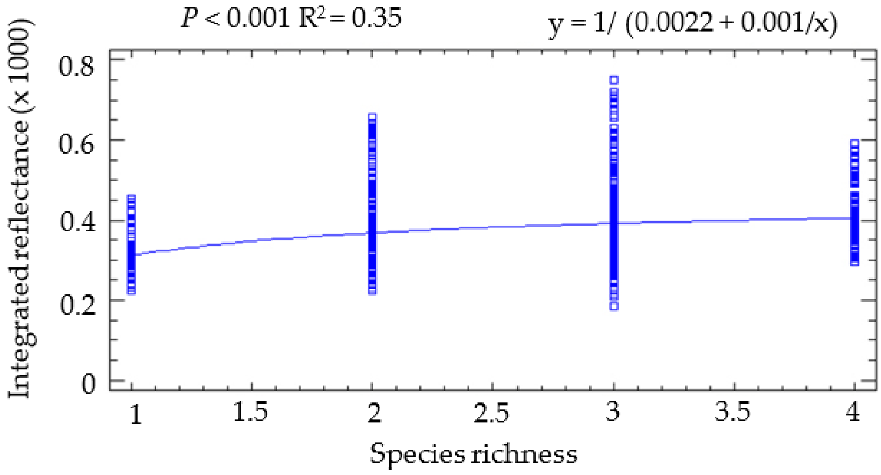

Supplementary Figure S2. Correlation curve of the integrated reflectance versus species richness in rain exclusion condition.

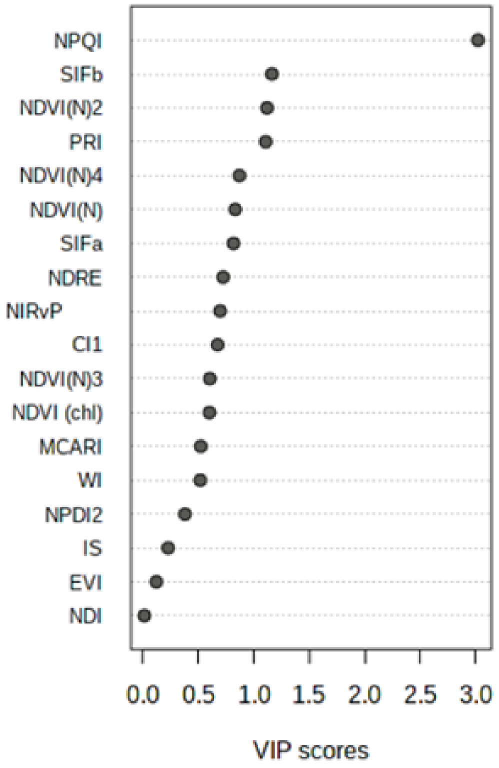

Supplementary Figure S3. Important features (VIP) identified by PLS-DA from the 20 selected vegetation indices.
